# Quercetin Protects Obesity-Induced Hypothalamic Inflammation by Reducing Microglia-Mediated Inflammatory Responses via HO-1 Induction

**DOI:** 10.3390/nu9070650

**Published:** 2017-06-23

**Authors:** Jihyeon Yang, Chu-Sook Kim, Thai Hien Tu, Min-Seon Kim, Tsuyoshi Goto, Teruo Kawada, Myung-Sook Choi, Taesun Park, Mi-Kyung Sung, Jong Won Yun, Suck-Young Choe, Jee Hye Lee, Yeonsoo Joe, Hye-Seon Choi, Sung Hoon Back, Hun Taeg Chung, Rina Yu

**Affiliations:** 1 Department of Food Science and Nutrition, University of Ulsan, Ulsan 44610, Korea; yjh6516@hanmail.net (J.Y.); ppar75@ulsan.ac.kr (C.-S.K.); tuthai_hien2000@yahoo.com (T.H.T.); sychoe@ulsan.ac.kr (S.-Y.C.); lljh2000@ulsan.ac.kr (J.H.L.); 2 Division of Endocrinology and Metabolism, University of Ulsan College of Medicine, Seoul 05505, Korea; mskim@amc.seoul.kr; 3 Graduate School of Agriculture, Kyoto University, Uji, Kyoto 611-0011, Japan; tgoto@kais.kyoto-u.ac.jp (T.G.); fat@kais.kyoto-u.ac.jp (T.K.); 4 Department of Food Science and Nutrition, Center for Food and Nutritional Genomics Research, Kyungpook National University, Daegu 41566, Korea; mschoi@knu.ac.kr; 5 Department of Food and Nutrition, Yonsei University, Seoul 03722, Korea; tspark@yonsei.ac.kr; 6 Department of Food and Nutrition, Sookmyung Women’s University, Seoul 04310, Korea; mksung@sookmyung.ac.kr; 7 Department of Biotechnology, Daegu University, Gyeongbuk 38453, Korea; jwyun@daegu.ac.kr; 8 Department of Biological Science, University of Ulsan, Ulsan 44610, Korea; jcantibody@ulsan.ac.kr (Y.J.); hschoi@ulsan.ac.kr (H.-S.C.); shback@ulsan.ac.kr (S.H.B.); chung@ulsan.ac.kr (H.T.C.)

**Keywords:** obesity, inflammation, hypothalamus, microglia, quercetin

## Abstract

Obesity-induced hypothalamic inflammation is characterized by activation of microglia, which are resident macrophages of the central nervous system, and is implicated in the derangement of energy homeostasis, metabolic complications, and neurodegenerative diseases. Quercetin, a naturally occurring flavonoid, is known to protect against oxidative stress and inflammation-related metabolic complications. Here, we demonstrate that quercetin reduces obesity-induced hypothalamic inflammation by inhibiting microglia-mediated inflammatory responses, and the beneficial action of quercetin is associated with heme oxygenase (HO-1) induction. Quercetin markedly reduced the production of inflammatory mediators (monocyte chemoattractant protein (MCP)-1, interleukin (IL-6), IL-1β, nitric oxide) by microglia stimulated with saturated fatty acid palmitate and/or lipid-laden microglia-conditioned medium. Quercetin also upregulated the expression of HO-1 in palmitate-treated lipid-laden microglia, and the actions of quercetin against microglia activation accompanied by IκBα degradation were abolished by a HO-1 inhibitor. Moreover, quercetin supplementation reduced the levels of inflammatory cytokines and microglia activation markers in the hypothalamus of high fat diet (HFD)-fed obese mice, which was accompanied by upregulation of HO-1. These findings indicate that quercetin suppresses microglia-mediated inflammatory responses via the induction of HO-1, and hence protects against obesity-induced hypothalamic inflammation.

## 1. Introduction

Obesity-induced low-grade systemic inflammation, which occurs mainly in peripheral tissues such as adipose tissue and liver, is characterized by increased infiltration of macrophages and production of inflammatory mediators [1]. A growing body of evidence shows that obesity-induced inflammation occurs not only in the peripheral tissue, but also in areas of the central nervous system (CNS) such as the hypothalamus [2]. The hypothalamus regulates energy balance by integrating peripheral and neuronal signals of satiety and nutrients, and thus regulates food intake, energy expenditure, and metabolism [3]. Intriguingly, the mediobasal hypothalamus has fenestrated capillaries, which are lack of blood brain barrier (BBB) [4], and thus cells in the area can easily sense and respond to peripheral signals [5]. Moreover, it is likely that the BBB permeability can be altered by a high fat-feeding obese condition [6]. These suggest that under obese/diabetic condition the peripheral excessive nutrients and hormones (e.g., fatty acid, glucose, or leptin) may affect the hypothalamic environment. Studies have shown that obesity induces hypothalamic inflammation accompanied by neuronal damage [7], and this is implicated in leptin/insulin resistance, disrupted energy homeostasis, and neurodegenerative diseases [8,9]. Thus, factors controlling obesity-related deleterious hypothalamic inflammation may have the potential to protect against hypothalamic dysfunction and neuro-metabolic complications.

Microglia, which are the resident macrophages of the CNS, account for 12–15% of the glial cells in the brain [10]. Microglial cells have many functions and molecular characteristics similar to peripheral tissue macrophages, and play important roles as immunocompetent phagocytic cells [11]. Microglia in the CNS are relatively quiescent under normal conditions, but respond to stimuli such as infection, injury, and pathogens by phagocytosing cellular debris or bacteria. Moreover, they play an important role in host defense and tissue repair by secreting inflammatory cytokines and chemokines such as tumor necrosis factor alpha (TNFα), interleukin (IL)-6, IL-1β, and monocyte chemoattractant protein-1 (MCP-1), reactive oxygen intermediates, and proteinases [12]. Although microglia are essential to host defense and tissue repair in the central nervous system [13], prolonged over-activation of microglia cells under acute or chronic pathological conditions causes inflammation-mediated neuronal damage and dysfunction [14]. Notably, obesity and/or high-fat feeding have been shown to trigger hypothalamic inflammation and neuronal injury [15], and the inflammatory response was accompanied by activation of microglia and cell proliferation [16]. More recently, inhibition of high fat diet (HFD)-induced microglia activation or proliferation was shown to prevent hypothalamic and peripheral inflammation, leading to reduced food intake and adiposity [17]. Among various peripheral factors, increased levels of circulating free fatty acids under obese conditions are likely critical to increased microglia-mediated inflammatory responses via activation of the toll-like receptor 4 (TLR4)-mediated nuclear factor-kappa B (NF-κB) pathway [18,19,20,21,22]. Given that activated microglia-derived inflammatory cytokines mediate neuronal apoptosis, whereas inhibition of microglia activation is neuroprotective [23], controlling microglia-mediated inflammatory responses may be a useful strategy against obesity-induced hypothalamic inflammation. 

Heme oxygenase (HO-1), an antioxidant enzyme that catalyzes the oxidative degradation of heme to biliverdin and carbon monoxide [24], plays a key role in defense mechanisms against oxidative damage and inflammation [25]. HO-1 is considered to be a protective mechanism in various inflammatory neurological conditions: expression of HO-1 in microglia can be induced by various oxidative stress-inducing pathological conditions [26], and the induction of HO-1 is considered to protect against neuronal damage [27]. Several naturally occurring flavonoid compounds including curcumin and resveratrol in fruits and vegetables and n-3 polyunsaturated fatty acids in oily fish have the potential to ameliorate microglia-mediated inflammatory responses [28,29]. Quercetin is a polyphenolic flavonoid found in many dietary sources (e.g., onion, apple, tomato, grape) that has previously been shown to exert protective effects against obesity-induced peripheral inflammation of the liver and adipose tissue [30,31,32,33,34]. Moreover, quercetin attenuates lipopolysaccharide-induced inflammatory responses in microglia [35,36]. The anti-inflammatory actions of quercetin are associated with the inhibition of inflammatory cytokines, inflammatory receptor expression, and their signaling pathways in the peripheral tissue [33] and the cells [35,36]. Although it has been shown that quercetin increases HO-1 expression [35,36,37,38] and protects against-oxidant induced cellular dysfunction [37,38], it remains unclear whether quercetin has the potential to suppress microglia-mediated hypothalamic inflammation under obese conditions and if the effects of quercetin are associated with HO-1.

In this study, we demonstrate that quercetin inhibited inflammatory responses in microglia treated with saturated fatty acid and/or lipid-laden microglia-conditioned medium in vitro, mimicking obese conditions. The protective actions of quercetin were abolished by HO-1 inhibitor. Moreover, quercetin supplementation reduced microglia activation markers and inflammatory cytokines in the hypothalamus of HFD-fed obese mice, and this was associated with upregulation of HO-1. These findings suggest that quercetin suppresses microglia-mediated inflammatory responses via the induction of HO-1, and hence protects against obesity-induced hypothalamic inflammation. 

## 2. Materials and Methods

### 2.1. Cell Cultures and Treatments

BV2 microglia cells were obtained from the Meta-Inflammation Basic Research Laboratory (University of Ulsan, Ulsan, Korea). Cells were cultured in RPMI1640 (Gibco BRL, Grand Island, NY, USA) supplemented with 10% fetal bovine serum (Gibco) at 37 °C under 5% CO_2_. BV2 microglia cells and/or primary microglia [39] were seeded at a density of 2 × 10^5^ cells/mL in 24-well plates and treated with 5–10 μM quercetin for 2 h. The cells were then stimulated with free fatty acid (FFA) for 6 h or 48 h. Quercetin (Jena Bioscience, Jena, Germany) was dissolved in dimethyl sulfoxide (DMSO) and diluted in serum-free medium. The final concentration of DMSO in the medium was <0.05%. The FFA, palmitate (Sigma-Aldrich, St. Louis, MO, USA), was dissolved in ethanol and conjugated with bovine serum albumin (BSA) (30 μM) at a 10:1 molar ratio prior to use. The HO-1 activity inhibitor, Tin Protoporphyrin IX dichloride (TinPPIX) (Santa Cruz, CA, USA), was dissolved in 25 mM NaOH and diluted in serum-free medium. N1 hypothalamic neuronal cells (mHypo E-N1) were obtained from the Appetite Regulation Laboratory, Asan Institute for Life Science (University of Ulsan College of Medicine, Seoul, Korea). The cells were cultured in Dulbecco’s modified Eagle medium (DMEM) (Gibco) supplemented with 10% fetal bovine serum (Gibco) at 37 °C and 5% CO_2_. 

### 2.2. Animal Experiment

All animal care and procedures were conducted according to the protocols and guidelines approved by the University of Ulsan Animal Care and Use Committee (LYN-15-010). Seven-week-old male B6/SJL mice were obtained from the Jackson laboratory (The Jackson Laboratory, Bar Harbor, ME, USA). Mice were maintained under specific pathogen-free conditions at 22 °C and given access to food and water ad libitum. To examine the effects of quercetin on obesity-induced hypothalamic inflammation, mice were adapted for 1 week, then randomly divided into three dietary groups (*n* = 5 per group) and fed for 8 weeks on (1) a low fat diet (LFD; 10% calories from fat; Research Diet Inc., New Brunswick, NJ, USA); (2) a high-fat diet (HFD; 60% of calories from fat; Research Diets Inc.); and (3) the HFD supplemented with 0.05% quercetin (HFD + 0.05% Que; 0.05 g quercetin/100 g diet; approximately 50 mg/kg body weight/day). The dose of quercetin was adapted from our previous study [31,32] which showed a beneficial effect against obesity-induced inflammation in peripheral tissues.

### 2.3. Adipose Tissue Conditioned Medium (ATCM) Collection

ATCM was prepared from obese mice fed a high-fat diet (HFD, 60% of calories from fat; Research Diets Inc. or a regular diet (RD, control ATCM). To prepare the ATCM, mice were adapted for 1 week and then randomly divided into two dietary groups and fed for 8 weeks. Adipose tissue was isolated into phosphate buffered saline (PBS), minced into ~5–10 mg pieces, and then placed into nylon mesh, after which the tissue was washed with buffer containing 0.15 M NaCl, 10 mM KH_2_PO_4_, and 5 mM glucose [40]. Adipose tissue was then placed into culture dishes containing DMEM HG (0.2% Fungizone) media. The cultures were subsequently placed in a cell incubator at 37 °C and allowed to equilibrate for 72 h. After that, ATCM were collected and stored at −70 °C for subsequent analyses and treatment.

### 2.4. Oil Red O Staining

To determine lipid accumulation, cells were fixed with 10% formaldehyde for 10 min at room temperature, washed with 60% isopropyl alcohol, and then stained for 10 min with 0.21% Oil red O (Sigma) in 60% isopropanol. After washing with distilled water, the stained cells were observed under a microscope (Olympus, Tokyo, Japan). The stained lipid droplets were subsequently quantified with an ELISA reader (Molecular Devices, Sunnyvale, CA, USA) at 490 nm after extraction with isopropanol.

### 2.5. Triglyceride Measurement

The cellular content of triglyceride (TG) was measured using a TG enzymatic assay kit (Asan Pharmaceuticals, Seoul, Korea). The cellular protein concentration was determined using a bicinchoninic acid protein assay kit (Thermo Scientific, Pittsburgh, PA, USA). The cellular TG was normalized to the cellular protein content.

### 2.6. Preparation of Lipid-Laden Microglia-Conditioned Medium (LL-M-CM)

To prepare the LL-M-CM, microglia were treated with or without palmitate for 48 h, then cultured for 24 h without palmitate, after which the media were collected.

### 2.7. Nitric Oxide (NO) Assay

The amount of nitrite in the culture medium was measured by the Griess reaction. Briefly, 100 μL of medium was mixed with an equal volume of Griess reagent on a 96-well plate. The absorbance at 570 nm was then measured after 10 min using an ELISA reader and the amount of nitrite was calculated from a NaNO_2_ standard curve.

### 2.8. Measurement of Cytokine Levels

The levels of cytokine in culture supernatants were measured by enzyme-linked immunosorbent assays (ELISA). The assays were conducted using an OptEIA mouse MCP-1, IL-1β set (BD Bioscience Pharmingen, San Diego, CA, USA), and a mouse IL-6 Quantikine ELISA kit (R&D Systems, Minneapolis, MN, USA) according to the manufacturer’s instructions. 

### 2.9. Western Blot Analysis

The cells were lysed in lysis buffer (150 mM NaCl, 50 mM Tris-HCl (pH 7.4), 50 mM NaF, 10 mM Na_4_P_2_O_7_, 1 mM Ethylendiaminetetraacetic acid, 1% IGEPAL) supplemented with protease inhibitors cocktail (Sigma). Protein concentrations of the lysates were determined by BCA protein assay reagents (Pierce). Equal amounts of protein (5–10 μg) were subjected to western blot analysis using polyclonal antibodies to HO-1 (Enzo Life Sciences, Farmingdale, NY, USA), IκBα (Santa-Cruz), and β-actin (Sigma-Aldrich). Protein bands were detected using an enhanced chemiluminescence western blotting detection kit (PerkinElmer, Waltham, MA, USA). Band intensities were quantified by densitometry using the Image J program.

### 2.10. Quantitative Real-Time PCR (qRT-PCR)

Total RNA extracted from cultured cells or the hypothalamus was reverse transcribed into cDNA using M-MLV reverse transcriptase (Promega, Madison, WI, USA). Real-time PCR amplification of the cDNA was performed with a SYBR premix Ex Taq kit (TaKaRa Bio Inc., Foster, CA, USA) using a Thermal Cycler Dice (TaKaRa Bio Inc., Shiga, Japan). All reactions were performed by subjecting the samples to the same conditions: initial denaturation at 95 °C for 10 s, followed by 45 cycles of 95 °C for 5 s and 60 °C for 30 s. The results were analyzed using the real-time system TP800 software (TaKaRa Bio Inc., Shiga, Japan) and all values for genes were normalized to those of the housekeeping gene, β-actin. The primers used in the analysis are listed in Table 1.

### 2.11. Cell Viability Assay

Cell viability was measured by a 3-(4,5-dimethyl-2-thiazolyl)-2,5-diphenyl-2H-tetrazolium bromide (MTT) assay (Sigma). For the assay, 5 × 10^3^ cells/well of mHypo E-N1 cells were seeded on 96-well flat plates. mHypo E-N1 cells were treated with LL-M for 72 h, after which 100 µL of media and 10 µL of MTT solution were added. After 4 h of incubation, colored crystals of formazan were dissolved in 200 µL of dissolving solution, after which the optical density (OD) was read on a multiwell scanning spectrophotometer (ELISA reader) at 570 nm.

### 2.12. Statistical Analysis

The results are presented as the means ± standard error of the mean (SEM). Statistical analyses were performed using a one-way analysis of variance (ANOVA) with Turkey’s multiple comparison and/or Student’s *t* test using GraphPad Prism 5 (San Diego, CA, USA). Differences were considered to be significant at *p* < 0.05.

## 3. Result

### 3.1. Microglia Activation by Adipose Tissue-Conditioned Medium

**Figure 1:**
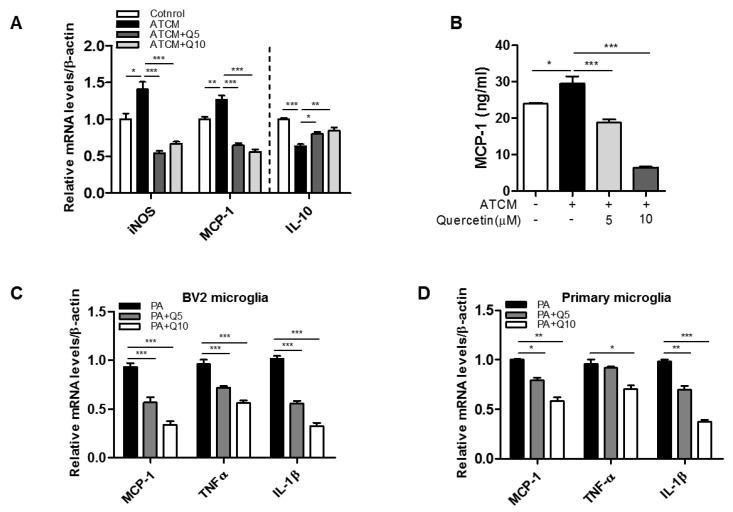
Effect of quercetin on microglia activation treated with adipose tissue conditioned medium (ATCM) or free fatty acid (FFA). Microglia (BV2) were treated with ATCM in the presence or absence of quercetin (5, 10 μM) for 24 h. (**A**) The transcription levels of iNOS, MCP-1, and IL-10 were measured by RT-PCR analysis; (**B**) The levels of MCP-1 in the conditioned-medium were measured by ELISA; (**C**,**D**) Microglia were treated with FFA (palmitic acid) (300 μM) for 6 h. The transcription levels of inflammatory cytokines (MCP-1, TNFα, IL-1β) were measured by RT-PCR analysis. Data are the means ± SEM of three independent experiments performed in triplicate. * *p* < 0.05, ** *p* < 0.01, *** *p* < 0.001 compared with control.

To test whether adipose-mediated factors induce microglia activation, we exposed BV2 microglia cells to ATCM. ATCM from obese mice significantly upregulated the transcript level of MCP-1 and inducible nitric oxide synthase (iNOS) mRNA in BV2 cells (Figure 1A), while it downregulated the transcript level of the anti-inflammatory cytokine, IL-10 (Figure 1A). Quercetin significantly decreased the transcript levels of iNOS and MCP-1 and increased the transcript levels of IL-10 in the BV2 microglia. Quercetin also markedly decreased the levels of MCP-1 released (Figure 1B). Additionally, it significantly reduced palmitate-induced inflammatory responses in BV2 microglia and primary microglia isolated from the hypothalamus by downregulating the transcript levels of MCP-1, TNFα, and IL-1β (Figure 1C,D). These results indicate that quercetin may reduce microglia-mediated inflammatory responses under obese conditions.

### 3.2. Increased Inflammatory Responses in Lipid Accumulation in Microglia

**Figure 2:**
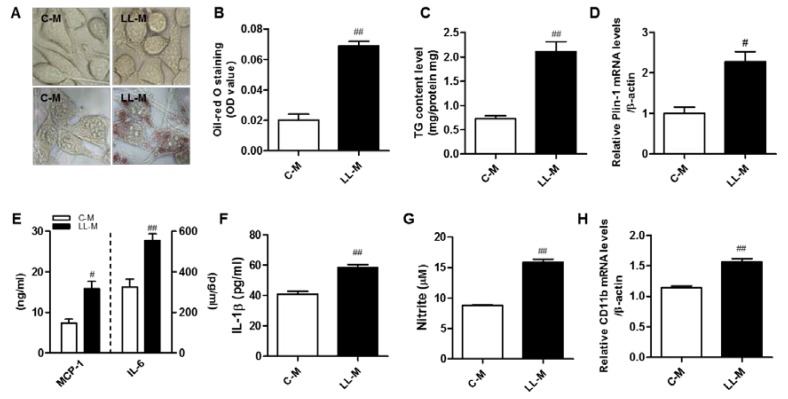
Increased inflammatory responses of lipid-laden microglia. (**A**) Microglia were treated with palmitic acid (300 μM) for 48 h and cells were visualized by microscopy. Using Oil red O, lipids were stained red; (**B**) Oil red O was extracted using isopropanol, and was quantified at 490 nm; (**C**) Triglyceride contents in FFA-treated microglia; (**D**) The transcription level of Plin-1 was measured by RT-PCR analysis; (**E**,**F**) The levels of MCP-1, IL-6, and IL-1β in the conditioned medium were measured by ELISA; (**G**) The amounts of nitrite in the conditioned medium were measured by the Griess method; (**H**) The transcription level of CD11b was measured by RT-PCR analysis. Data are the means ± SEM of three independent experiments performed in triplicate. # *p* < 0.005, ## *p* < 0.001 compared with the control.

Obesity results in excess accumulation of lipids in peripheral cells such as adipocytes, hepatocytes, and macrophages, which are accompanied by increased inflammatory responses and metabolic dysregulation. We investigated whether microglia can accumulate lipid droplets under FFA-rich condition, mimicking obesity. To accomplish this, we treated BV2 microglia with FFA (300 µM palmitate) for 48 h, then stained the samples with Oil red O. As shown in Figure 2A,B, Oil red O staining revealed that the accumulation of cytosolic lipid droplets in BV2 microglia increased significantly following FFA treatment. The intracellular TG contents also increased significantly in BV2 microglia treated with FFA (Figure 2C). We also found that transcript level of Plin-1, a marker of lipid droplet protein, was upregulated (Figure 2D). More importantly, we found that the lipid-laden microglia elicited inflammatory phenotypes. Specifically, the production of MCP-1, IL-6, and IL-1β protein were markedly increased in the lipid-laden microglia when compared with the control (Figure 2E,F), and the NO release was higher in the lipid-laden microglia than in the control (Figure 2G). We also confirmed that the level of CD11b transcript, a marker for microglia activation, was upregulated in the lipid-laden microglia cells (Figure 2H). These results suggest that the accumulation of lipid droplets in microglia following FFA treatment results in microglia activation, increasing their inflammatory responses.

### 3.3. Effect of Quercetin on the Inflammatory Responses of Lipid-Laden Microglia

**Figure 3:**
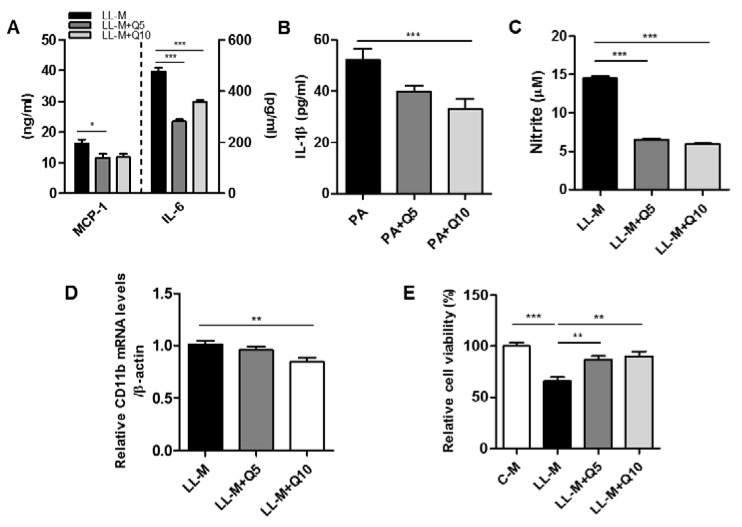
Effect of quercetin on the inflammatory response of lipid-laden microglia. Microglia were pretreated with quercetin (5, 10 μM) for 2 h, and then treated with palmitic acid (300 μM) for 48 h. (**A**,**B**) The levels of MCP-1, IL-6, and IL-1β in the conditioned-medium were measured by ELISA; (**C**) The amounts of nitrite in the conditioned-medium were measured by the Griess method; (**D**) The transcription levels of CD11b were measured by RT-PCR analysis; (**E**) The cell viability was evaluated by MTT assay. Data are the means ± SEM of three independent experiments performed in triplicate. * *p* < 0.05, ** *p* < 0.01, *** *p* < 0.001 compared with control.

We next examined whether quercetin suppresses the inflammatory responses in lipid-laden microglia. As shown in Figure 3A–C, quercetin treatment significantly reduced the levels of inflammatory cytokine (MCP-1, IL-6, IL-1β) release and inhibited the NO released from lipid-laden microglia. Moreover, CD11b transcript levels were downregulated in the lipid-laden microglia treated with quercetin (Figure 3D). Subsequently, we found that the lipid-laden microglia-conditioned medium reduced the viability of mHypo E-N1 cells, and that quercetin protected against microglia-conditioned medium-induced mHypo E-N1 cells injury (Figure 3E). 

### 3.4. Effect of Quercetin on HO-1 Induction and Inflammatory Signaling in Lipid-Laden Microglia

**Figure 4:**
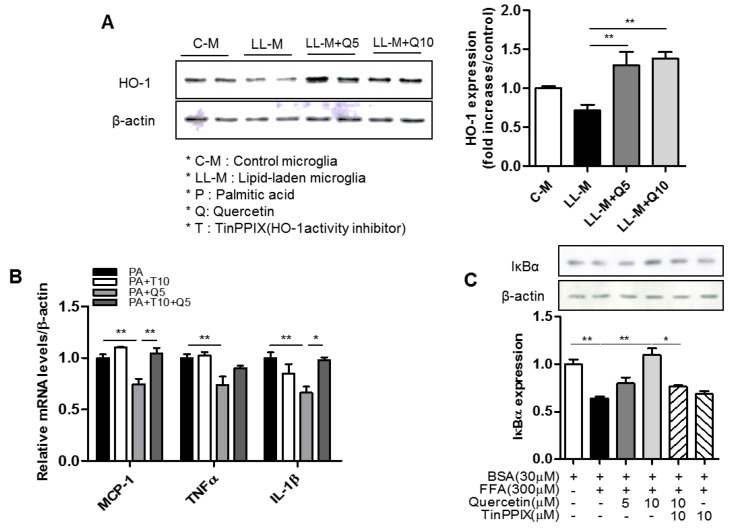
Effect of quercetin on inflammatory response and heme oxygenase (HO-1) inhibition in lipid-laden microglia. Microglia were pretreated with quercetin (5, 10 μM) for 2 h, then treated with palmitic acid (300 μM). (**A**) The expression of HO-1 protein was analyzed by western blotting with antibody against HO-1 protein; (**B**) The transcription levels of inflammatory cytokines (MCP-1, TNFα, IL-1β) were measured by RT-PCR analysis; (**C**) The expression of IκBα protein was analyzed by western blotting with antibody against IκBα protein. Data are the means ± SEM of three independent experiments performed in triplicate. * *p* < 0.05, ** *p* < 0.01 compared with control.

HO-1 is an antioxidant enzyme that plays a key role in defense mechanisms against oxidative damage and inflammation. Therefore, we tested the potential involvement of HO-1 in the inhibitory effects of quercetin against microglia-mediated inflammatory responses. First, we confirmed that quercetin treatment upregulated HO-1 protein in the FFA-treated BV2 microglia cells (Figure 4A). We next found that quercetin reduced levels of inflammatory cytokines (TNFα, IL-1β, MCP-1) in the FFA-stimulated BV2 microglia cells, but that these effects were blunted by the HO-1 inhibitor, TinPPIX (Figure 4B). Because activation of NF-κB plays a key role in the induction of major inflammatory mediators, we subsequently examined whether quercetin affects FFA-mediated activation of NF-κB in BV2 microglia. As shown in Figure 4C, western blotting revealed that quercetin inhibited FFA-induced degradation of IκBα, suggesting that it could suppress NF-κB activation. Moreover, TinPPIX significantly blunted the effects of quercetin (Figure 4C). 

### 3.5. Effect of Quercetin on Hypothalamic Inflammation in HFD-Fed Obese Mice

**Figure 5:**
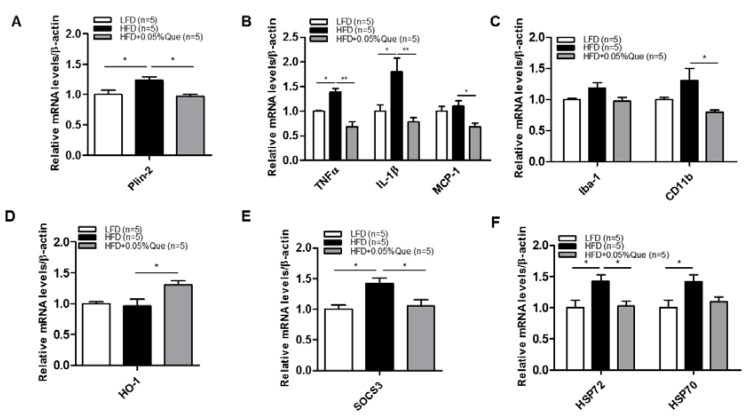
Effect of quercetin supplementation on obesity-induced hypothalamic inflammation in obese mice fed a high fat diet (HFD). B6/SJL mice were fed a low fat diet (LFD, 10% of calories from fat), high fat diet (HFD, 60% of calories from fat), or HFD + 0.05% quercetin for 8 weeks. The hypothalamuses were then isolated from LFD (*n* = 5), HFD (*n* = 5), and quercetin fed HFD (*n* = 5). (**A**) Expression of Plin-2; (**B**) inflammatory cytokine (TNFα, IL-1β, and MCP-1) genes; (**C**) markers of microglia activation (Iba-1 and CD11b); (**D**) expression of HO-1; (**E**) SOCS3; (**F**) HSP72 and HSP70 genes in hypothalamus from LFD (*n* = 5), HFD (*n* = 5), and HFD + 0.05% quercetin (*n* = 5) fed mice. Values are means ± SEM * *p* < 0.05, ** *p* < 0.01, significantly different from LFD control (LFD vs. HFD), HFD control (HFD vs. HFD + 0.05% quercetin).

To examine the effects of quercetin on hypothalamic inflammation in vivo, we generated obese mice by feeding them a HFD with or without quercetin. We found that HFD significantly upregulated the transcript level of the lipid droplet marker, Plin-2 (Figure 5A). Furthermore, quercetin supplementation significantly downregulated transcript levels of inflammatory cytokines/chemokine (TNFα, IL-1β, MCP-1) (Figure 5B), as well as markers of microglia/activation (Iba-1 and CD11b) transcripts in the hypothalamus of HFD-fed obese mice (Figure 5C). Indeed, quercetin significantly upregulated HO-1 transcript in HFD-fed obese mice (Figure 5D). Finally, we observed that quercetin significantly decreased neuronal damage markers such as heat shock protein (HSP)72 and HSP70, and downregulated suppressor of stress signaling-3 (SOCS3), a negative regulator of leptin signaling (Figure 5E,F). 

## 4. Discussion and Conclusions

Under obese condition, hypertrophic adipose tissue releases large amounts of non-esterified saturated fatty acid (FFA), mostly palmitate, and thus increases their levels in circulation, leading to activation of inflammatory signaling pathway and insulin resistance. Studies have shown that FFA can pass through the BBB [41] and stimulate neuronal and glia cells [42], and indeed activation of microglia is observed in the hypothalamus of HFD-feeding/obese mice [43]. In this study, we for the first time found that obese ATCM-treated microglia upregulated levels of inflammatory mediators (MCP-1, TNFα, and IL-1β) at the transcript and/or protein levels, and FFA treatment did the same as well, indicating that adipose tissue-derived peripheral factors such as FFA may enhance microglia-mediated inflammatory responses. Of note, we observed that prolonged treatment of microglia with saturated fatty acid palmitate resulted in the accumulation of lipid droplets in the microglia. The lipid-laden microglia released inflammatory mediators (MCP-1, IL-6, IL-1β, NO) with the upregulation of CD11b, an activation marker, and IκBα degradation reflecting activation of the NF-κB pathway, indicating that the lipid-laden microglia elicit inflammatory phenotype. Importantly, quercetin significantly reduced the production of inflammatory mediators from the ATCM-treated and/or FFA-stimulated microglia, and the lipid-laden microglia. Together, these findings indicate that quercetin has the potential to reduce the microglia-mediated inflammatory responses under the excessive adipose tissue-derived factors such as FFA in obese condition.

To clarify the mechanism of quercetin action, we tested the association of quercetin action with HO-1, which is a rate-limiting enzyme that plays a critical role in attenuating oxidative stress, inflammation, and metabolic dysregulation [26,44,45]. In this study, along with the inhibitory action of quercetin on the inflammatory responses in the lipid-laden microglia, we observed that quercetin treatment markedly upregulated HO-1 protein expression in the lipid-laden microglia. To determine if HO-1 is responsible for the quercetin action, we tested the effects of the HO-1 inhibitor TinPPIX on the quercetin action against the microglia-mediated inflammatory responses. We found that the inhibitory effects of quercetin on FFA-stimulated microglia activation were blunted by the HO-1 inhibitor, TinPPIX. Moreover, the inhibitory action of quercetin against microglia activation, which was mediated by suppression of IκBα degradation and led to NF-κB inactivation, was also blunted by the HO-1 inhibitor. These findings indicate that the activation of HO-1 by quercetin may compromise microglia-mediated inflammatory response via NF-κB interruption. 

Growing evidence shows that the quercetin can cross the BBB [46,47] and has the potential to reduce neuroinflammation and neurodegenerative disorders [48]. Hence, we subsequently examined the inhibitory action of quercetin on microglia-mediated inflammatory responses in vivo. Using obese mice fed an HFD, we confirmed that quercetin supplementation reduced the transcript levels of inflammatory cytokines (TNFα, IL-1β, MCP-1) in the hypothalamus of obese mice fed an HFD, and also reduced the transcript level of the lipid droplet marker Plin2. Additionally, quercetin supplementation markedly reduced the transcript levels of macrophage activation markers (Iba-1 and CD11b) in the hypothalamus of obese mice fed an HFD, and this was accompanied by upregulation of HO-1. These findings indicate that quercetin suppresses hypothalamic lipid-laden microglia-mediated inflammatory responses under obese conditions, and that the beneficial actions of quercetin are associated with its effect on HO-1 induction. Of note, along with increased neuronal damage markers such as HSP72/HSP70, we observed that the level of SOCS3 transcript, a major negative regulator of leptin signaling [49,50], was upregulated in the inflamed hypothalamus of HFD-fed obese mice, and that these changes were suppressed in the hypothalamus of quercetin-supplemented obese mice. Taken together, these findings suggest that quercetin may be beneficial in preventing microglia-mediated hypothalamic inflammation, and it may improve leptin resistance in obese condition.

**Figure 6:**
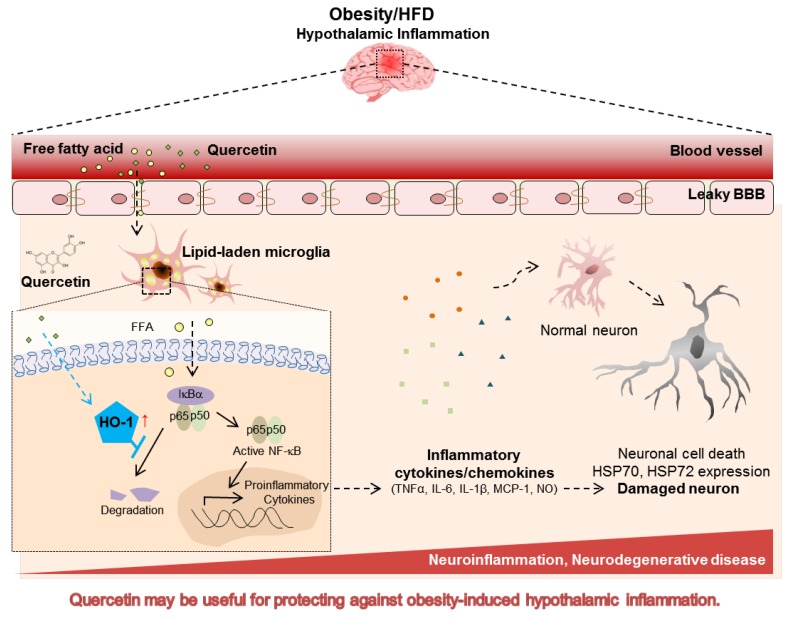
Effect of quercetin on microglia-mediated hypothalamic inflammation in obese conditions. Hypothalamic microglia accumulates lipid droplets under fatty acid-rich obese conditions. Lipid-laden microglia release inflammatory cytokines, leading to microglia activation and neuronal damage. Quercetin reduced the lipid-laden microglia mediated inflammatory responses and this was accompanied by upregulation of HO-1. Quercetin may be useful for protecting against obesity-induced hypothalamic inflammation and related diseases. BBB, blood brain barrier.

In conclusion, we demonstrated that quercetin reduces the lipid-laden microglia-mediated inflammatory responses through inhibition of NF-κB activation, and that the quercetin action was abolished by the HO-1 inhibitor. Moreover, quercetin supplementation in HFD-fed obese mice reduced hypothalamic inflammatory markers, and this was accompanied by the upregulation of HO-1 (Figure 6). These findings suggest that quercetin suppresses lipid-laden microglia-mediated inflammatory responses via the induction of HO-1, and hence protects against obesity-induced hypothalamic inflammation. Accordingly, quercetin may be a useful dietary factor for preventing obesity-induced hypothalamic inflammation.

## Figures and Tables

**Table 1:** Mouse primers used for quantitative real-time (qRT)-PCR.

Primer Name	Forward Primer Sequence	Reverse Primer Sequence
*iNOS*	CAAGCTGAACTTGAGCGAGGA	TTTACTCAGTGCCAGAAGCTGGA
*MCP-1*	GCATCCACGTGTTGGCTCA	CTCCAGCCTACTCATTGGGATCA
*IL-10*	GCCAGAGCCACATGCTCCTA	GATAAGGCTTGGCAACCCAAGTAA
*TNFα*	AAGCCTGTAGCCCACGTCGTA	GGCACCACTAGTTGGTTGTCTTTG
*IL-1β*	TCCAGGATGAGGACATGAGCAC	GAACGTCACACACCAGCAGGTTA
*Plin-1*	GAGAGAGCCATGACGCACAGA	TGTGTACCACACCACCCAGGA
*CD11b*	CCACTCATTGTGGGCAGCTC	GGGCAGCTTCATTCATCATGTC
*Plin-2*	GGCTACGACGACACCGATGA	GGACAGTCTGGCATGTAGTCTGGA
*Iba-1*	TGGTCCCCCAGCCAAGA	CCCACCGTGTGACATCCA
*HO-1*	TGCAGGTGATGCTGACAGAGG	GGGATGAGCTAGTGCTGATCTGG
*SOCS3*	GATTCACCCAGGTGGCTACA	CTCGGACCTACTGACCGAGA
*HSP72*	CAGAGGCCAGGGCTGGATTA	ACACATGCTGGTGCTGTCACTTC
*HSP70*	CGCTCGAGTCCTATGCCTTCA	GGCACTTGTCCAGCACCTTC
*β-actin*	CATCCGTAAAGACCTCTATGCCAAC	ATGGAGCCACCGATCCACA
